# Evaluation of the effect of *Heterorhabditis bacteriophora* (HP88) on *Stomoxys calcitrans* (Linnaeus, 1758) larvae (Diptera: Muscidae) in sugarcane bagasse ash

**DOI:** 10.29374/2527-2179.bjvm002123

**Published:** 2023-10-02

**Authors:** Américo de Castro Monteiro, Ana Caroline Ferreira de Souza, Danielle Pereira da Silva, Graziele Calixto Souza, Isadora Luiza Alves Costa, João Luiz Lopes Monteiro, Melissa Carvalho Machado do Couto Chambarelli, Avelino José Bittencourt

**Affiliations:** 1 Veterinarian, MSc. Programa de Pós-Graduação em Ciências Veterinárias (PPGCV), Departamento de Parasitologia Animal (DPA). Instituto de Veterinária (IV), Universidade Federal Rural do Rio de Janeiro (UFRRJ). Seropédica, Seropédica, RJ, Brazil.; 2 Veterinarian Autonomus. Rio de Janeiro, RJ, Brazil.; 3 Agronomist, DSc. Programa de Pós-Graduação em Agronomia (POSAGRO), Departamento de Engenharia Agrícola (DEA). Centro de Ciências Agrárias (CCA), Universidade Federal de Roraima (UFRR). Cauamé, Boa Vista, RR, Brazil.; 4 Veterinarian, DSc. DPA, IV, UFRRJ. Seropédica, Seropédica, RJ, Brazil.; 5 Veterinarian, DSc. Departamento de Medicina e Cirurgia Veterinária (DMCV), IV, UFRRJ. Seropédica, Seropédica, RJ, Brazil.

**Keywords:** ash, biological control, stable fly, nematodes, cinzas, controle biológico, mosca dos estábulos, nematoides

## Abstract

The purpose of this study was to evaluate the effect of the EPN *Heterorhabditis bacteriophora* HP88 on *Stmoxys. calcitrans* larvae in sugarcane bagasse ash. Groups of 10 stable fly larvae were placed in Petri dishes containing filter paper and bagasse ash. Concentrations of 50, 150 and 250 EPNs/larva of *S. calcitrans* in four milliliters of distilled water were added to each plate. In the control group contained only distilled water, without EPNs. The bioassay had three replications and was maintained at 27 ± 1°C and 70-80% relative humidity. It was observed that mortality rate in all treated groups was significantly higher than in the control group (26,6%). The mortality rate in the presence of 50 EPNs/larva (46,6%) was lower than in 150 EPNs/larva (76,3%), which in turn was lower than 250 EPNs/larva group (93,3%). It was verified by analysis of variance and regression that there was a linear pattern of mortality, that is, the higher the EPNs/larva concentration, the higher the larval mortality. It was concluded that EPN *H. bacteriophora* HP88 was capable of infecting and causing mortality of stable fly larvae in sugarcane bagasse ash.

## Resumo

O objetivo deste estudo foi avaliar o efeito do NEP *Heterorhabditis bacteriophora* HP88 sobre larvas de *Stomoxys calcitrans* em cinzas de bagaço de cana-de-açúcar. Grupos de 10 larvas da mosca dos estábulos foram depositadas em placas de Petri contendo papel filtro e cinzas. Foram adicionadas concentrações de 50, 150 e 250 NEPs/larva de *S. calcitrans* em cada placa. No grupo controle não havia NEPs, somente água destilada. O bioensaio teve três repetições e foi mantido em 27 ± 1°C e 70-80% de umidade relativa. Observou-se que a mortalidade em todos os grupos tratados foi significativamente superior à do grupo controle (26,6%). A taxa de mortalidade na presença de 50 NEPs/larva (46,6%) foi menor do que em 150 NEPs/larva (76,3%), que por sua vez foi menor do que no grupo 250 NEPs/larva (93,3%). Verificou-se pela análise de variância e de regressão que houve um padrão linear de mortalidade, ou seja, quanto maior a concentração de NEPs/larva, maior a mortalidade larval. Conclui-se que o NEP *H. bacteriophora* HP88 foi capaz de infectar e causar mortalidade das larvas da mosca dos estábulos em cinza de bagaço de cana e que aparentemente este subproduto não interfere negativamente na ação deste NEP.

## Introduction

*Stomoxys calcitrans* is a hematophagous dipteran, commonly known as the stable fly, which can parasitize a variety of animal species, including humans (Bittencourt & Moya-Borja, 2000). Parasitism by the stable fly, a hematophagous insect, is not only a biological and mechanical vector of various pathogens but also highly stressful to livestock due to its painful bites, causing significant economic losses (Grisi et al., 2014).

The extraction of sugarcane juice generates a large amount of bagasse, a biomass that is a very important source of energy. About 95% of all sugarcane bagasse produced in Brazil is burned in boilers to generate energy, producing bagasse ash as residue (Zancaner & Santos, 2013). In addition to ash, the sugar and alcohol mills generates byproducts such as filter cake, vinasse and sugarcane bagasse, which are used to as fertilizers in sugarcane plantations. The stable fly uses these byproducts as substrates for its development, causing outbreaks of these arthropods in areas surrounding these mills (Souza et al., 2021). Since 1973, through the work of Nakano et al. (1973), the relationship between the occurrence of S. *calcitrans* and by-products of sugar and alcohol production in these mills has been known. According to Corrêa et al. (2013), even though it does not affect the productivity of sugarcane crops, this fly is able to develop widely in sugarcane fields fertilized by sugarcane byproducts. The very method used for fertirrigation, which is sprinkling vinasse on the sugarcane fields, favors the formation of suitable areas for the development of the fly during the entire sugarcane harvest period (Bittencourt, 2012). The relationship between overpopulations of *S. calcitrans* and sugar cane by-products is no longer a focal problem, and tends to follow the expansion of cane fields and farms (Dominghetti et al., 2015). The substrates available in the sugar and alcohol properties with potential to promote overpopulations of *S. calcitrans* are straw with vinasse, filter cake and bagasse ash (Corrêa et al., 2013).

In view of these insect resistance to chemical insecticides and the latter’s harmful effect on to the environment, alternative pest control methods are sought, including biological control, in which context entomopathogenic nematodes (EPNs) may play a useful role (Kaya & Gaugler, 1993). The pathogenic action of EPNs on arthropods is directly dependent on symbiotic bacteria that live inside it, bacteria of the genus *Photorhabdus*, in *Heterorhabditis* spp. (Burnell & Stock, 2000). The process of death of the host insect begins after the migration of EPNs to the hemocoel: the resulting release of mutualistic bacteria present in their intestines, which in their proliferation secrete lethal toxins, causes rapid sepsis (Hazir et al., 2003) in their hosts.

Studies involving stable fly and EPNs are still scarce in the literature, however, Leal et al. (2017) and Monteiro Sobrinho et al. (2021) showed promising results using *Heterorhabditis bacteriophora* HP88 and *H. baujardi* LPP7 against stable fly larvae. Monteiro Sobrinho et al. (2023), presented interesting results of EPNs controlling *S. calcitrans* larvae in sugarcane by-products, however, in this study there was no bagasse ash. The purpose of the present study was to evaluate the effect of the entomopathogenic nematode (EPN) *H. bacteriophora* (HP88) on third-instar larvae of *S. calcitrans* in sugarcane bagasse ash.

## Material and methods

The *Stomoxys calcitans* colony used in this study was reared on a laboratory benchtop (at 27 ± 1°C and 70-80% relative humidity - RH), using an adapted version of the method described by Macedo et al. (2005). The EPNs colony, in turn, was reared *in vivo* using the method described by Lindegren et al. (1993), which consisted in breeding and multiplication on *Galleria mellonella* (Lepidoptera: Pyralidae). The infective juveniles (IJs) were kept in a 40mL cell culture flask and stored in a Biochemical Oxygen Demand (BOD) temperature-controlled incubator (Eletrolab®, model EL 202/4) at 16 ± 1°C and 70-80% RH for less than a week. To calculate the doses used in this study, the IJs were counted in twelve aliquots of 10μL taken from an aqueous suspension of EPNs. After counting the IJs in the 12 aliquots, the highest and lowest numbers of EPNs/aliquot were discarded and the average number of IJs in the remaining 10 aliquots was calculated. Based on this calculation, the concentration of the suspensions was adjusted to IJs/mL (Taylor et al., 1998). Four groups of 10 third-instar *S. calcitrans* larvae were placed in Petri dishes containing two sheets of filter paper and five grams of sugarcane bagasse ash. Concentrations of 0, 50, 150 and 250 EPNs/larva of *S. calcitrans* in four milliliters of distilled water were added to each plate. The control group contained only sugarcane bagasse ash and distilled water, however without EPNs. The bioassay was monitored daily, with three replications. The plates were covered with plastic film and kept in a BOD incubator (Eletrolab®, model EL 212) at 25 ± 1°C and 70 ± 10% RH.

The data were subjected to analysis of variance, after which the results obtained underwent regression analysis using the statistical software SISVAR 5.O (Ferreira, 2011).

## Results

**Figure 1:**
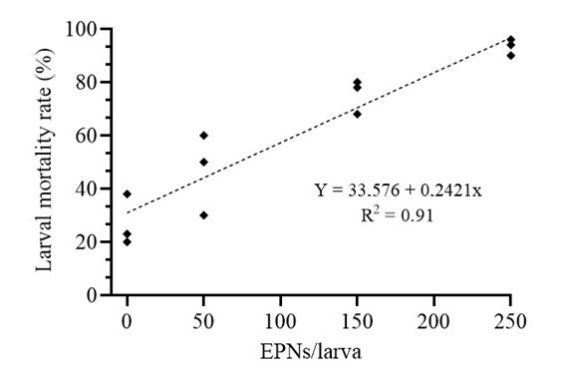
*Stomoxys calcitrans* larval mortality rate as a function of EPNs concentration.

All the treated groups with EPNs showed a significantly higher mortality rate than the control group (0 EPNs/larva) (26,6%). The mortality rate in the 50 EPNs/larva group (46,6%) was lower than in the 150 EPNs/larva group (76,3%), which in turn was lower than 250 EPNs/larva group (93,3%) (Figure 1). Analysis of variance and of regression revealed a linear pattern of mortality, the higher the EPNs/larva concentration, the higher the mortality rate.

## Discussion

Leal et al. (2017), tested the effect of the EPN *H. bacteriophora* on stable fly larvae, reported a 96.7% mortality rate at a concentration of 200 EPNs/larva. This mortality rate is similar than that achieved in the present study with 250 EPNs/larva (93,3%). However, Leal et al. (2017) did not test EPNs associated with sugarcane bagasse ash, which suggests that the substrate appears does not negatively affected the activity of EPNs at this concentration. This suggests that the effect of using sugarcane bagasse ash as a substrate could probably be offset by increasing the concentration of EPNs/larva. In addition, it may be necessary to increase the amount of water used for the free mobility of EPNs, since ash has properties that can absorb environmental moisture (Cacuro & Waldman, 2015), thereby limiting the mobility of EPNs. Monteiro Sobrinho et al. (2021) observed a mortality rate of 91,7% of *S. calcitrans* larvae at a concentration of 200 EPNs/larva in 48 hours of exposure of larvae to nematodes. Their result is similar than that achieved in the present study at 250 EPNs/larva (93,3%), even with the EPNs in contact with the fly larvae throughout the bioassay. However, it should be noted that the studies of Monteiro Sobrinho et al. (2021) and Leal et al. (2017) used no substrate that prevented the action of EPNs, only distilled water. In a study using *H. bacteriophora* HP88 on *S. calcitrans* larvae grown on filter cake substrate, Monteiro Sobrinho et al. (2016) reported a mortality rate of 76,6% when using a concentration of EPNs/larva. Their results are very similar to those achieved in our study at the same concentrations used by the aforementioned authors, 76.6% using 150 EPNs/larva. This indicates that, like filter cake, sugarcane bagasse ash negatively affects the activity of EPN, acting either as a barrier that prevents EPN from reaching the larvae, or because of the absorption of environmental water, thus hindering the movement of these organisms, which need a water layer to move (Grewal et al., 2001). Monteiro Sobrinho et al. (2023) reported that EPNs are effective on *S. calcitrans* larvae in a variety of sugarcane substrates, and sugarcane bagasse ash is a substantial substrate produced by sugar mills, and although it is produced in smaller quantities than vinasse, bagasse and sugarcane straw, it is also used for the fertilization of sugarcane fields, favoring the development of immature stages of the stable fly, which causes significant losses in livestock and in the sugar and alcohol industry (Corrêa at al., 2013).

## Conclusions

It was concluded that EPN *H. bacteriophora* HP88 was able to infect and kill stable fly larvae in sugarcane bagasse ash.

## References

[B001] Bittencourt A. J. (2012). Avaliação de surtos e medidas de controle ambiental de *Stomoxys calcitrans* (Diptera: Muscidae) na Região Sudeste do Brasil. Revista Brasileira de Medicina Veterinária.

[B002] Bittencourt A. J., Moya-Borja G. E. (2000). Flutuação sazonal de *Stomoxys calcitrans* em bovinos e equinos no município de Espírito Santo do Pinhal. Revista Universidade Rural. Série Ciências da Vida.

[B003] Burnell A. N. N. M., Stock S. P. (2000). *Heterorhabditis, Steinernema* and their bacterial symbionts-lethal pathogens of insects. Nematology.

[B004] Cacuro T. A., Waldman W. R. (2015). Cinzas da queima de biomassa: Aplicações e potencialidades. Revista Virtual de Química.

[B005] Corrêa E. C., Ribas A. C. A., Campos J., Barros A. T. M. (2013). Abundância de *Stomoxys calcitrans* (Diptera: Muscidae) em diferentes subprodutos canavieiros. Pesquisa Veterinária Brasileira.

[B006] Dominghetti T. F. S., Barros A. T. M., Soares C. O., Cançado P. H. D. (2015). *Stomoxys calcitrans* (Diptera: Muscidae) outbreaks: Current situation and future outlook with emphasis on Brazil. Revista Brasileira de Parasitologia Veterinária.

[B007] Ferreira D. F. (2011). Sisvar: A computer statistical analysis system. Ciência e Agrotecnologia.

[B008] Grewal P. S., De Nardo E. A. B., Aguillera M. M. (2001). Entomopathogenic nematodes: Potential for exploration and use in South America. Neotropical Entomology.

[B009] Grisi L., Leite R. C., Martins J. R., Barros A. T. M., Andreotti R., Cançado P. H. D., Léon A. A. P., Pereira J. P., Villela H. S. (2014). Reassessment of the potential economic impact of cattle parasites in Brazil. Revista Brasileira de Parasitologia Veterinária.

[B010] Hazir S., Kaya H. K., Stock P., Keskin N. (2003). Entomopathogenic nematodes (Steinernematidae and Heterorhabditidae) for biological control of soil pests. Turkish Journal of Biology.

[B011] Kaya H. K., Gaugler R. (1993). Entomopathogenic nematodes. Annual Review of Entomology.

[B012] Leal L. C. S. R., Monteiro C. M. O., Mendonça A. E., Bittencourt V. R. E. P., Bittencourt A. J. (2017). Potential of entomopathogenic nematodes of the genus *Heterorhabditis* for the control of *Stomoxys calcitrans* (Diptera: Muscidae). Revista Brasileira de Parasitologia Veterinária.

[B013] Lindegren J. E., Valero K. A., Mackey B. E. (1993). Simple *in vivo* production and storage methods for *Steinernema carpocapsae* infective juveniles. Journal of Nematology.

[B014] Macedo D. M., Chaaban A., Moya-Borja G. E. (2005). Desenvolvimento pós-embrionário de *Stomoxys calcitrans* (Linnaeus, 1758) (Diptera: Muscidae) criadas em fezes de bovinos tratados com diferentes avermectinas. Revista Brasileira de Parasitologia Veterinária.

[B015] Monteiro A. C., Costa I. L. A., Souza G. C., Leal L. C. S. R., Monteiro J. L. L., Chambarelli M. C. M. C., Bittencourt A. J. (2021). Infection and reinfection of *Stomoxys calcitrans* larvae (Diptera: Muscidae) by entomopathogenic nematodes in different times of exposure. Revista Brasileira de Parasitologia Veterinária.

[B016] Monteiro A. C., Leal L. C. S. R., Monteiro J. L. L., Chambarelli M. C. M. C., Bittencourt A. J. (2023). Evaluation *in vitro* of the virulence of two entomopathogenic heterorhabditid nematodes in the control of *Stomoxys calcitrans* (Diptera: Muscidae) larvae in byproducts of the sugar and alcohol industry. Revista Brasileira de Parasitologia Veterinária.

[B017] Monteiro A. C., Mendes C. O. F., Leal L. C. S. R., Bittencourt A. J. (2016). Virulência de *Heterorhabditis bacteriophora* cepa HP88 (Rhabditida: Heterorhabtidae) sobre larvas de *Stomoxys calcitrans* (Díptera: Muscidae) em dieta de torta de filtro. Revista Brasileira de Medicina Veterinária.

[B018] Nakano O., Paro J. R. L. A., Camargo A. H. (1973). Controle químico de adultos e larvas da mosca doméstica. O Biológico.

[B019] Souza T. F., Cançado P. H. D., Barros A. T. M. (2021). Attractivity of vinasse spraying to stable flies, *Stomoxys calcitrans* in a sugarcane area. Pesquisa Veterinária Brasileira.

[B020] Taylor D. B., Szalanski A. L., Adams B. J., Peterson 2nd R. D. (1998). Susceptibility of house fly (Diptera: Muscidae) larvae to entomopathogenic nematodes (Rhabditida: Heterorhabditidae, Steinernematidae). Environmental Entomology.

[B021] Zancaner M. G., Santos T. B. S. (2013). Cogeração: Ampliação da oferta de energia elétrica com a biomassa (bagaço da cana-de-açúcar). Revista Diálogos Interdisciplinares.

